# A Behaviourally Informed Approach to Reducing the Risk of Inadvertent Anti-doping Rule Violations from Supplement Use

**DOI:** 10.1007/s40279-023-01933-x

**Published:** 2023-10-06

**Authors:** Susan H. Backhouse

**Affiliations:** Carnegie School of Sport, Leeds Beckett University, Leeds, UK

## Abstract

For many reasons, athletes’ use of supplements is highly prevalent across sports and competitive levels, despite the risk of these products containing a substance on the World Anti-Doping Agency Prohibited List. Contravening anti-doping rules through supplement use could have serious consequences for competitive athletes (e.g., ineligibility from major competitions, loss of medals and funding) due to the principle of strict liability. Indiscriminate supplement use also poses a risk to athlete health. To reduce the possibility of ingesting a supplement containing prohibited substances, independent quality assurance and certification programs have been established (e.g., Informed Sport). However, these programs do not completely eliminate risk, leading to some anti-doping organisations promoting a *‘just say no’* to supplements stance. Yet, this approach can be problematic as a small number of supplements may be necessary for athletes to consume, in certain situations. Recognising that athletes will continue to use these heavily marketed products, this narrative review describes a theoretically underpinned and systematic approach to preventing inadvertent doping by considering the barriers to and enablers of athlete adherence to risk minimisation supplement use guidelines (RMSUG). By outlining a conceptual shift towards a behaviourally informed approach, this review serves to stimulate the development of multifaceted interventions to prevent inadvertent doping through supplement use. Recognising that risk-minimised supplement use involves a myriad of behaviours, the problem of inadvertent doping through supplement use is framed, and research appraised, through the lens of the Behaviour Change Wheel.

## Key Points

**Table:** 

Athletes’ use of supplements is highly prevalent across sports and competitive levels, despite the risk of these products containing a substance on the World Anti-Doping Agency Prohibited List.
A theoretically and behaviourally informed approach to synthesising the evidence base on the barriers to and enablers of adhering to risk minimisation supplement use guidance (RMSUG) is presented.
Future research should identify the intervention types and behaviour change techniques which would be suitable for addressing the factors identified to improve transdisciplinary implementation of comprehensive risk-minimised supplement strategies and reduce the risk of inadvertent doping in sport.

## Introduction

Across sports, countries and competitive levels the use of supplements is commonplace [1–17], with higher use at elite levels reported [6]. The most recent systematic review and meta-analysis targeting athletes reported an overall prevalence of supplement use of approximately 60% among various sporting populations [6]. Previous reviews have estimated the prevalence of supplement use among athletes ranges between 11 and 100%, depending on several factors including the level of competition, type of sport and the definition of supplement use [3, 17]. Although not universally defined, the International Olympic Committee (IOC) consensus statement on dietary supplements and the high-performance athlete defined supplements as “a food, food component, nutrient, or non-food compound that is purposefully ingested in addition to the habitually consumed diet with the aim of achieving a specific health and/or performance benefit” (p. 439) [2]. Further, the expert panel acknowledged that supplements can be found in pill, capsule, powder or liquid form and contain dietary ingredients (e.g., vitamins, minerals, amino acids, botanicals) that can affect the body [2]. These functional effects on the body can drive many athletes to use a variety of supplements to manage the physical and social demands of sport, and enhance their performance and recovery [2, 17, 17–20]. Practical reasons also prompt supplement use, including convenience, food availability and nutrient deficiencies [2, 3, 19].

As with the definition of the products themselves, there is no universal approach to regulation, with many different frameworks developed that largely reflect national and regional priorities and needs [21]. For example, in the USA, melatonin is regulated as a supplement, in Canada it is considered a natural health product and in Australia it is a prescription medicine [21]. Further, the supplement industry has expanded at an alarming rate, exceeding the capacity of government agencies to regulate the market and protect the consumer [22]. Consequently, many products are sold on exaggerated claims and questionable evidence of safety and efficacy [2, 22, 23], with few supplements underpinned by an established evidence base [2, 24, 25]. Given this challenging and incongruent landscape, athletes and their support personnel (ASP) need guidance and support to navigate the pseudoscience and dis/misinformation [22]. Helpfully, the Australian Institute of Sport (AIS) has developed the Sports Supplement Framework (ABCD Classification system) [25], which provides an education tool to rank types of supplements according to the scientific evidence that they can safely and practically contribute to an athlete’s nutritional goals. This framework highlights that the effectiveness and safety of many supplements on the continuously growing global market have not been scientifically proven. Some may even be harmful to health [26, 27], with an estimated 23,000 emergency department visits every year in the US attributed to adverse events related to supplement use [28].

Beyond the pseudoscience and dis/misinformation associated with the supplement industry [22], widespread use of supplements in sport is problematic when combined with the omnipresent risk of contamination and/or adulteration of supplements with prohibited substances [29–32], putting athletes in a vulnerable position of unintentionally breaking World Anti-Doping Agency (WADA) rules [33, 34] and experiencing negative health effects [26–28, 35–37]. Studies have also found supplement use to be a correlate and predictor of self-reported doping behaviour or intention/likelihood to dope [38–42]. Whilst the magnitude of anti-doping rule violations (ADRVs) associated with supplement use is not yet known [43], the risk of prohibited substances being present in supplements has persisted for decades [31]. This threat has therefore been deemed to be a “small but real problem facing athletes who compete in events governed by anti-doping rules” (p. 1) [44]. Although the scale of this problem is unknown, some estimations have been made. One review noted that approximately 6–9% of reported doping cases are the result of athletes ingesting supplements containing prohibited substances [45]. Given the lack of global incidence and prevalence monitoring of inadvertent ADRVs through supplement use, there have been calls for anti-doping regulators to review current data-gathering provision and information systems so that the scale of this problem can be more directly and accurately assessed [43].

Due to the increasing demand for high-quality, properly labelled supplements, several organisations now offer testing and quality assurance programs for supplements prior to retail distribution [46, 47] (e.g., Informed Sport, LGC, UK—https://www.informed-sport.com; NSF Certified for Sport, NSF International, USA—https://www.nsfsport.com; NZVT list, the Netherlands—https://www.dopingautoriteit.nl/nzvt) [46, 48]. For athletes, it is critical they have assurances that the supplements they are ingesting are free from WADA prohibited substances before use. However, purchasing supplements remains a threat to athletes as even third-party testing and assurance organisations offer no guarantees. This is because no certification body can test for every substance on the non-exhaustive and regularly updated WADA Prohibited List [47]. Furthermore, most supplements do not undergo third-party testing [46, 48]. Consequently, athletes have long been warned by national and international anti-doping organisations about the risks of violating anti-doping rules through supplement use, with risk mitigation and fear appeals framing the guidance offered by practitioners and policy makers alike [46].

For anti-doping organisations tasked with delivering anti-doping education programs, fear appeals and risk mitigation strategies are driven by the principle of strict liability. Strict liability is the keystone of the World Anti-Doping Code [49] (hereafter referred to as ‘the Code’), the core document that harmonises anti-doping policies, rules and regulations within sport organisations and among public authorities around the world. Underpinning Article 2 of the Code is the statement that it is an athlete’s personal duty to ensure that no prohibited substance enters his or her body [49]. This means that an athlete can be judged to have committed an ADRV whether or not the athlete intentionally or unintentionally used a prohibited substance, or was otherwise negligent or at fault. However, the potential unfairness to athletes that strict liability creates has been recognised in the field of anti-doping [50], and the Code now provides provisions for athletes to avoid the full implications of strict liability and reduce what would normally be a mandatory sanction if strict liability principles were invoked [50]. For example, in respect of prohibited substances (e.g., anabolic androgenic steroids), the full implications can be avoided if the athlete can first show, on the balance of probabilities, how the prohibited substance entered their system and can then go on to establish, to the comfortable satisfaction of the anti-doping panel, that they bore either (i) no fault or negligence or (ii) no significant fault or negligence for its presence [50]. Whilst the Code makes a provision for the avoidance of or a reduction in sanction if the athlete can satisfactorily establish how the substance unintentionally entered their system, ignorance is not accepted as an excuse. Further, historical cases of doping highlight that the athlete is typically blamed, punished and publicly shamed in a doping conviction [50, 51]. To help athletes comply with the anti-doping rules of sport, WADA underscores the need for them to have access to information and receive education prior to doping control testing [49, 52]. The WADA has also placed greater emphasis on the role and responsibility of ASP within the Code in order to better recognise their influence on (anti-)doping behaviours [49].

Over the years, athletes have experienced a range of consequences for unintentionally violating the anti-doping rules, including detrimental physical and mental health effects, ineligibility from competition, and loss of funding or medals. For example, British 100-m sprinter CJ Ujah and his relay team were stripped of their Tokyo 2020 silver medals after Ujah tested positive for two prohibited substances (ostarine and S-23) which are selective androgen receptor modulators (SARMS) [53, 54]. Although the Athletic Integrity Unit and WADA were satisfied that Ujah ingested a contaminated supplement, he was unable to demonstrate that he was entitled to any reduction in the applicable period of ineligibility based on his level of fault [53]. Ujah had not followed established global guidance of only using batch-tested supplements [54].

The principle of strict liability interacts with a growing and vigorously marketed industry that is poorly regulated [21, 26]. Under these conditions, competitive athletes (as consumers) and ASP can find themselves in a vulnerable and risky position in regard to consuming supplements. The consequence of supplements being classified as a subcategory of food in many countries is that manufacturers do not need to provide evidence of product safety and efficacy compared, for example, with medications [26]. Moreover, supplements are easily purchased by the consumer without prescription or healthcare professional intervention [21]. Additional safety issues may arise from mega dosing, due to consumers’ belief that it can produce greater benefits than taking the daily recommended dose [55]. It is within the context of a complex adaptive supplement system that a prohibitive approach to supplement use has framed anti-doping organisation programmes [46], accompanied by ‘food first’ appeals (promoting a focus on conventional food and drinks). However, it is important to acknowledge that whilst a ‘food first’ approach is a goal of an evidence-based sports nutrition plan [25], some supplements can play a small but valuable role in an evidence-based sports nutrition plan [2, 19, 24, 25]. Consequently, there is growing recognition that ‘food first’, but not always ‘food only’ [19] is a more pragmatic and evidence-based strategy for athletes (when supported by qualified professionals [19]).

**Fig. 1:**
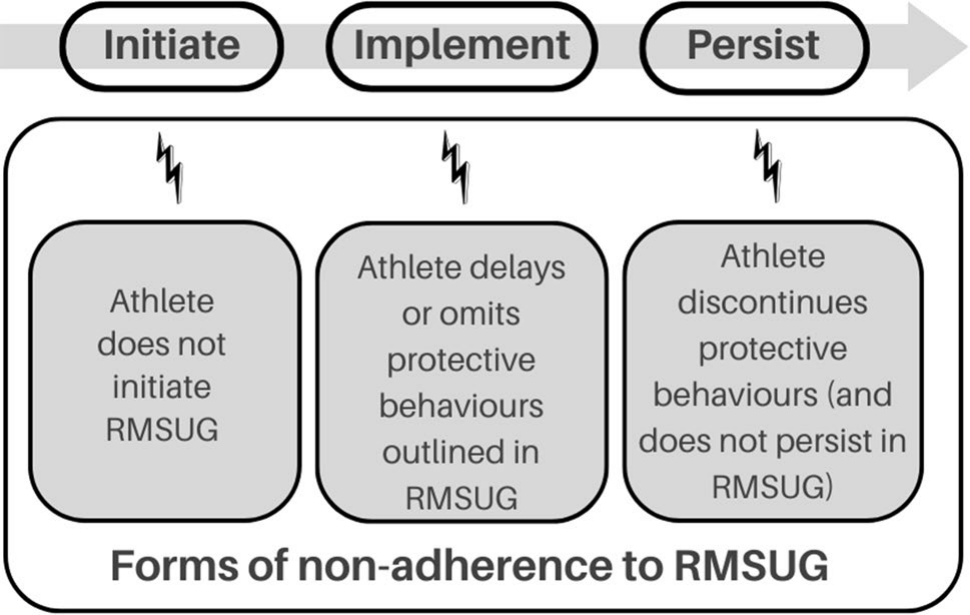
Forms of non-adherence to risk minimisation supplement use guidance (RMSUG)

Close and colleagues [19] proposed six reasons why a food-only approach may not be optimal for athletes, including the assertion that some nutrients are difficult to obtain in sufficient quantities in the diet, or may require excessive energy intake and/or consumption of other nutrients and some foods may be difficult to consume immediately before, during, or immediately after exercise [19]. Therefore, where the risk–benefit analysis [2, 56] supports the use of a supplement, it is critical that athletes adhere to risk minimisation supplement use guidance (RMSUG) (e.g., [2, 56]). Such guidance outlines the importance of athletes only using evidence-based supplements that they need and which present minimal risk of containing a WADA prohibited substance (with risk reduced due to third-party testing) to lessen the likelihood of inadvertently committing an ADRV. Within this guidance, athletes are also prompted to record and monitor their supplements to assist in establishing no significant fault or negligence. Non-adherence to RMSUG can lead to serious consequences for athletes. Therefore, this narrative review provides a contemporary perspective by proposing a conceptual and pragmatic shift towards a behaviourally informed approach to reducing the risk of committing an ADRV through adherence to RMSUG. Adherence has been defined as the extent to which a person’s behaviour (e.g., only ingesting supplements that are third-party tested) corresponds with agreed recommendations (e.g., from a professional body or organisation) [57], such as the International Olympic Committee (IOC) *Consensus Statement on Dietary Supplements and the High-Performance Athlete* [2] and British Dietetics Association (BDA) *Sports Nutrition Group Nutritional Supplement Position Statement* [56]. It is recognized that there is a spectrum of adherence to RMSUG spanning initiation, implementation and persistence (Fig. 1). However, adherence as the global term will be considered in this review.

## Designing Interventions to Foster Adherence to RMSUG Using the Behaviour Change Wheel

Extensive knowledge and understanding of the physiological and metabolic effects of supplements amounts to little if we cannot persuade athletes to adhere to RMSUG (e.g., only using the supplements they need and that have a robust scientific evidence-base for use, in doses that are required and that carry minimal risks of harm to health and/or committing an ADRV). Behavioural science plays an especially important role in understanding adherence, which is complex and driven by multiple behaviours. Adherence in this context refers to the extent to which an athlete’s supplement use behaviour corresponds with RMSUG (e.g., from a professional body or organisation) [57], such as the IOC [2], BDA [56], AIS [25] or US Anti-Doping Agency [58]. The pragmatic use of supplements that have passed a risk (cost)–benefit analysis of being effective, safe and permitted for use is promoted by global sports bodies in a bid to provide information to assist them to make informed decisions [2]. The guidance may differ slightly across organisations, but the content typically aligns with the IOC Consensus Statement flow chart to guide informed decision making and reduce risk of ADRVs through supplement use [2]. The purpose of IOC consensus statements is to inform, support and guide sports medicine clinical practice [59] and this guidance may not be reaching audiences that would benefit from the information provided [59] (e.g., athletes, coaches, anti-doping organisations). Whilst WADA have not published their own guidance on preventing inadvertent doping in sport through supplement use, WADA’s Medical Director was a co-author of the IOC Consensus Statement which signals WADA’s support of a risk mitigation strategy. However, more could be done by global sports bodies to raise awareness of RMSUG. Further, it is important to recognise that as multiple supplement use position statements/stands and guidance documents currently exist, it is perhaps unclear to athletes and ASP which guidance document should be followed. To ensure clarity and avoid any potential confusion, the development of a global consensus statement regarding the behaviours expected of athletes (and ASP) to reduce risk of inadvertent doping through supplement use would benefit the sporting community.

**Fig. 2:**
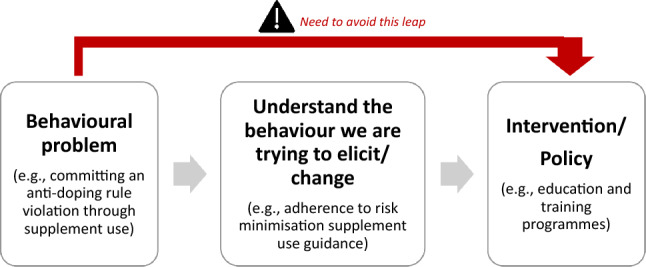
The importance of behavioural science

Developing our understanding of adherence to RMSUG allows us to deliver evidence-based interventions to reduce the risk of athletes committing an ADRV through supplement use. However, empirical research regarding the possibilities for athletes to adhere to such guidance is scant, as is the case for athletes’ compliance with anti-doping rules [52]. Furthermore, the focus of the current system on ‘catching the cheats’ means that little attention or support is afforded to those upholding the integrity of sport and navigating the complex anti-doping landscape, with athletes left to their own devices to cope with the fear of tarnishing their reputation with an inadvertent rule violation [60], exacerbated by the dopogenic environment [61]. This absence of evidence notwithstanding, research underscores the importance of taking a behaviourally informed approach rather than jumping straight from the behavioural problem to the intervention/policy (Fig. 2) [62].

**Fig. 3:**
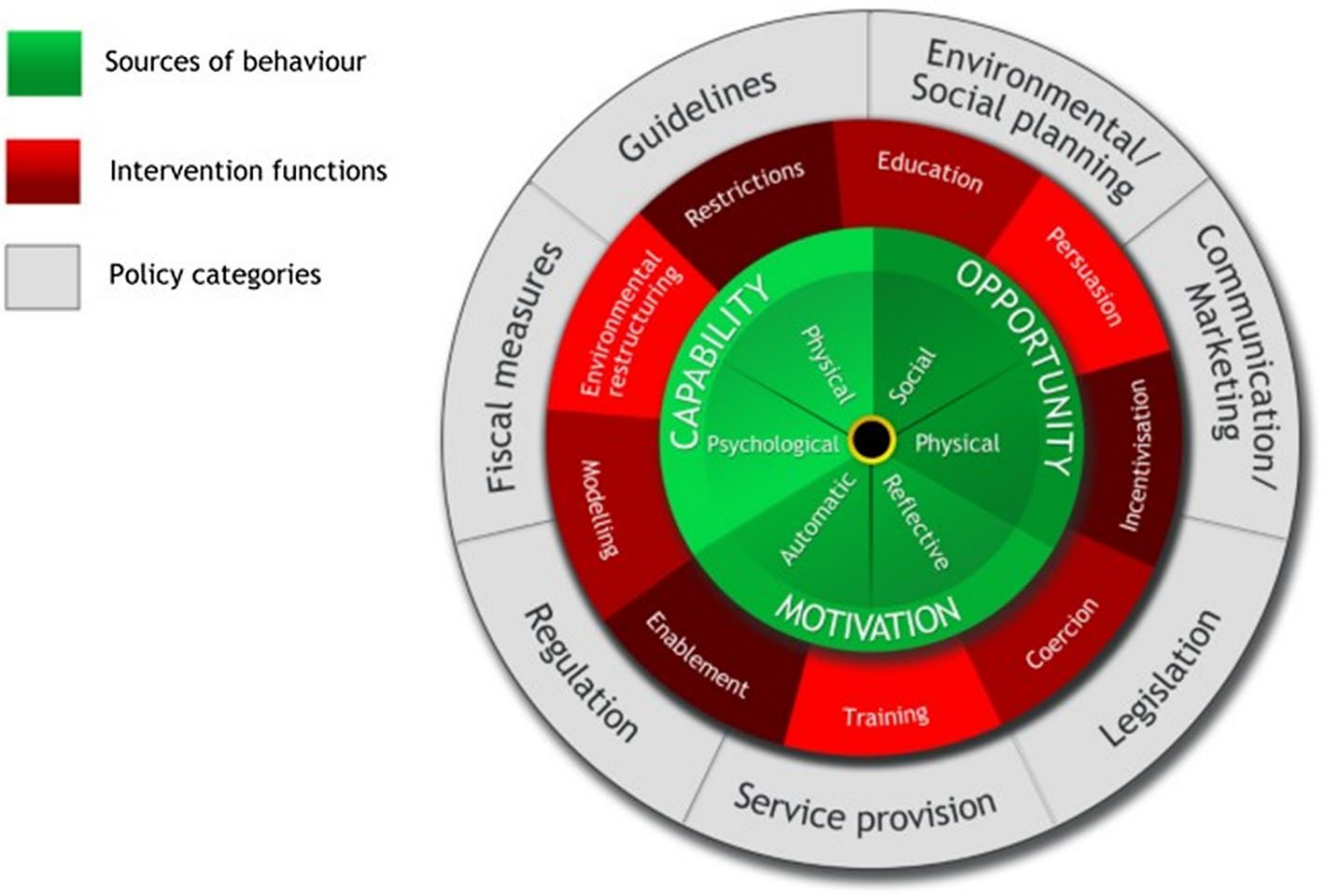
The Behaviour Change Wheel and the Capability, Opportunity, Motivation Model of Behaviour (COM-B). Reproduced with permission from Michie et al. [62]

The Behaviour Change Wheel (BCW) (Fig. 3) is an implementation model developed from synthesising 19 different behavioural change frameworks [62, 63]. It was developed in 2011 to help practitioners from across disciplines identify appropriate interventions or policies when trying to encourage adoption of a particular behaviour. The intervention design method described in the BCW guide is separated into three tasks for intervention designers: (1) understand the behaviour; (2) identify intervention options; (3) identify content and implementation options. The inner green hub represents the factors that influence any behaviour (capability, opportunity and motivation); the red circle shows the range of types of intervention (e.g., restrictions, environmental restructuring, education, modelling and training); and the grey outer circle shows policy options that can be used to deliver interventions (e.g., environmental/social planning, communication/marketing, legislation and guidelines). In addition to offering an integrated theoretical framework, and step-by-step guide to developing, delivering and evaluating behavioural interventions targeting behavioural problems, the BCW has been designed to be used by anyone, from any discipline, making application of theory more accessible to researchers and practitioners with a lack of in-depth knowledge of behaviour change theories [62].

This narrative review draws upon the extant literature to illustrate the first task of the BCW, comprising four steps. Step 1: Define the problem in behavioural terms; Step 2: Select the target behaviour; Step 3: Specify the target behaviour; Step 4: Identify what needs to change to achieve the target behaviour.

## Defining the Problem of Committing an Antidoping Rule Violation Through Supplement Use in Behavioural Terms

**Fig. 4:**
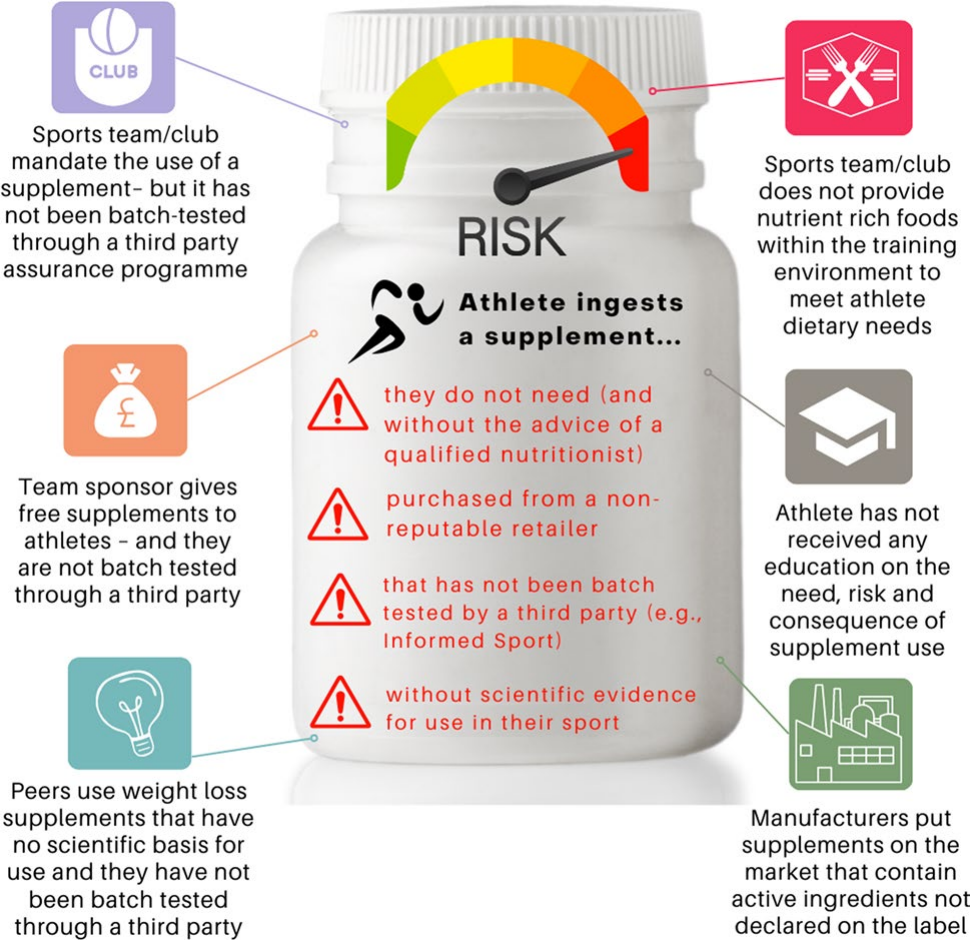
Behaviour as part of a system: the example of risky supplement use

Supplement use behaviours occur within complex dynamic systems, and Fig. 4 provides an example of the interdependence of behaviours related to ‘risky’ supplement use (i.e., athletes not following RMSUG). For example, this figure highlights the influence of significant others around the athlete, and widens the lens through which we view the problem; moving beyond athlete blame and shame in relation to inadvertent ADRVs associated with supplement use. The proposed theoretical approach helps us to recognise the behavioural problem of committing an ADRV through supplement use as a consequence of environmental conditions and opportunities (termed the *dopogenic* environment [61]) and not just the result of poor personal choice (Fig. 4).

As highlighted in the introduction, the risk of an inadvertent ADRV through the use of supplements remains a problem for ‘at-risk’ groups such as athletes competing in events governed by anti-doping rules [44]. If we are to prevent this problem and reduce the behavioural risk to athletes (and ASP), it is important that we clearly define all the relevant behaviours that enable adherence to RMSUG. As yet, a list of all the potential behaviours (and who needs to perform them) that may be relevant to tackling the problem of committing an ADRV from supplement use is still to be developed. This process is important to undertake, via a transdisciplinary approach, as the BCW emphasises the importance of consideration of each behaviour in terms of (i) the impact of changing the behaviour (what difference will it make?), (ii) the likelihood of changing the behaviour (to what extent can the behaviour be easily changed?) and (iii) any spillover effect (will changing the behaviour positively or negatively influence other behaviours?) [62]. By considering these criteria, the field will be better placed to make pragmatic and evidence-informed decisions on which behaviours to target through intervention (to reduce the risk of inadvertent doping through supplement use). At the same time, it is important to be specific about which behaviour(s) we are trying to change so that we can be more focused in our efforts to understand these behaviours. Finally, we need to acknowledge that it may be more effective to intervene intensively on one or two target behaviours and build on small successes than to attempt to change too much too soon [62, 63].

## Selecting the Target Behaviour

**Fig. 5:**
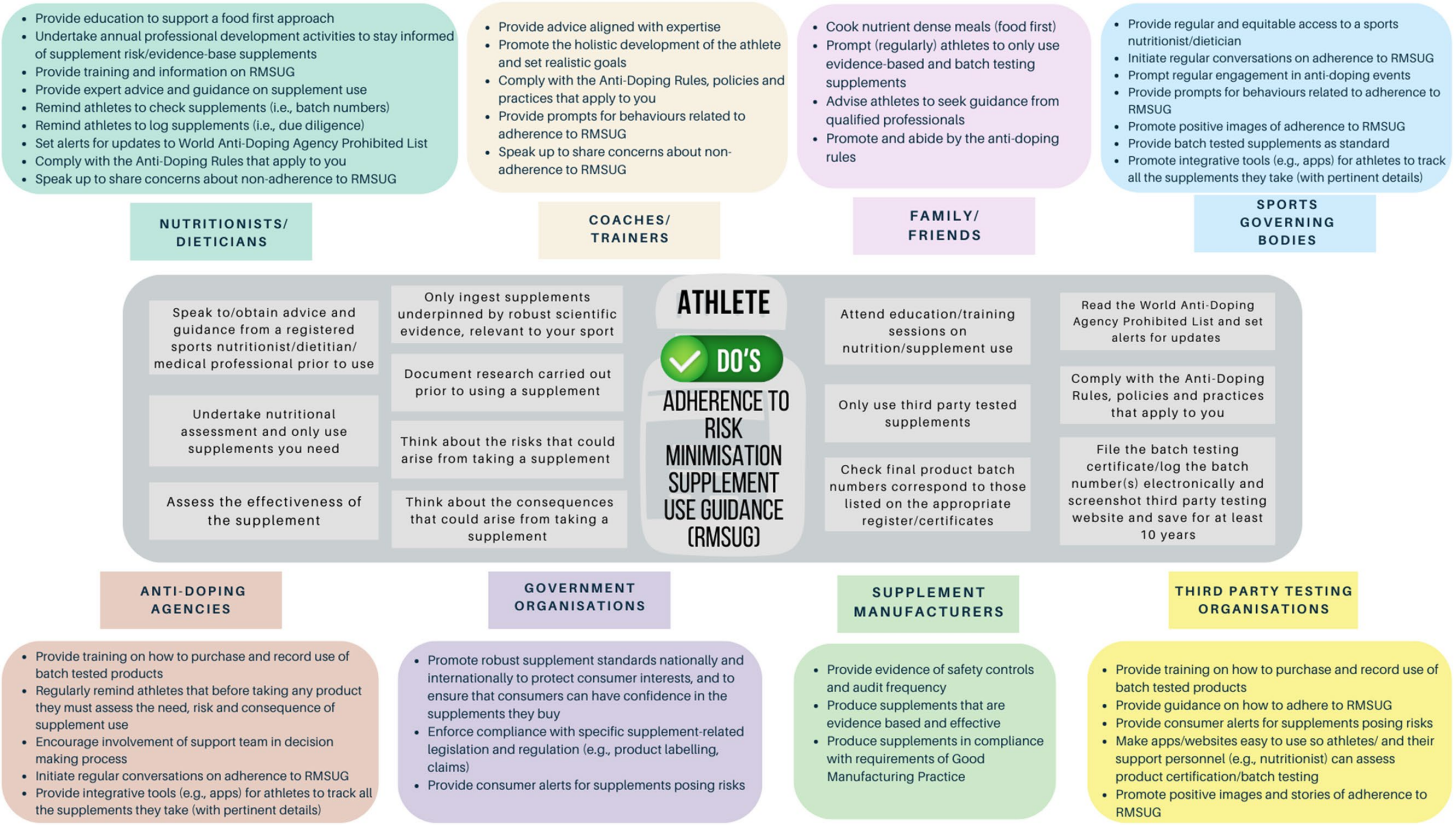
Preliminary behavioural matrix of actors involved in athlete adherence to risk minimisation supplement use guidelines (RMSUG)

Taking the behavioural problem of committing an unintentional ADRV through supplement use, a desk-based review of relevant supplement use decision-making guidelines (e.g., BDA Sports Nutrition Specialist Group Nutritional Supplement Position Statement [56]; IOC Consensus Statement [2]; AIS Position Statement [25]) underpinned the development of a preliminary conceptual matrix of behaviours relevant to adherence to RMSUG by competitive athletes (Fig. 5). It is important to emphasise that this behavioural matrix is not exhaustive or final. Instead, it serves as a stimulus for further development through transdisciplinary consensus building processes involving multiple stakeholders. Nonetheless, this initial matrix highlights that some of the behaviours relate directly to the competitive athlete consuming the supplement, whereas other behaviours involve other influential people, including ASP with whom the athlete interacts (e.g., coach, nutritionist), and people from the supplement industry and sports system. There will be other actors whose behaviours are not yet represented on this matrix that will also need due consideration and addition in the future.

If we focus on the competitive athlete (where current guidance/education is most targeted), national anti-doping organisations typically reinforce the importance of assessing the *need*, *risk* and *consequence* of supplement use [46]. Taking a behavioural perspective, and synthesising the guidance with action in mind, athletes are cautioned to *behave* in a number of different ways. For example, they should (i) obtain the advice and guidance of a well-informed sports nutrition professional to undertake a nutritional assessment before decisions regarding supplement use are made; (ii) assess the effectiveness of a supplement (e.g., related to a targeted event and its conditions, the specific individual, the combination with other performance strategies) and only use supplements that have a robust scientific basis for use; (iii) have a plan to monitor their supplement use; (iv) use supplements that have been through a recognised third-party supplement certification programme (e.g., Informed Sport, NSF) independent of the product manufacturers; (v) cross reference batch numbers to ensure the supplement they intend to ingest is indeed certified; (vi) record all the supplements being used (including research prior to use, product names and batch numbers); (vii) report all of their documented products and batch information on their doping control form (if they are subject to a test). Adherence to these actions help to minimise the risk of inadvertent doping and provide evidence that athletes were not at fault/negligent if an adverse analytical finding arises through supplement use (i.e., due diligence). To undertake these behaviours as a matter of habit and routine, conscious effort and vigilance by athletes is required (with support from their team). The multiple behaviours outlined in the preliminary behavioural matrix (Fig. 5) underscore the importance of targeting specific behaviours and target groups through research and intervention design.

## Identifying What Needs to Change

**Fig. 6:**
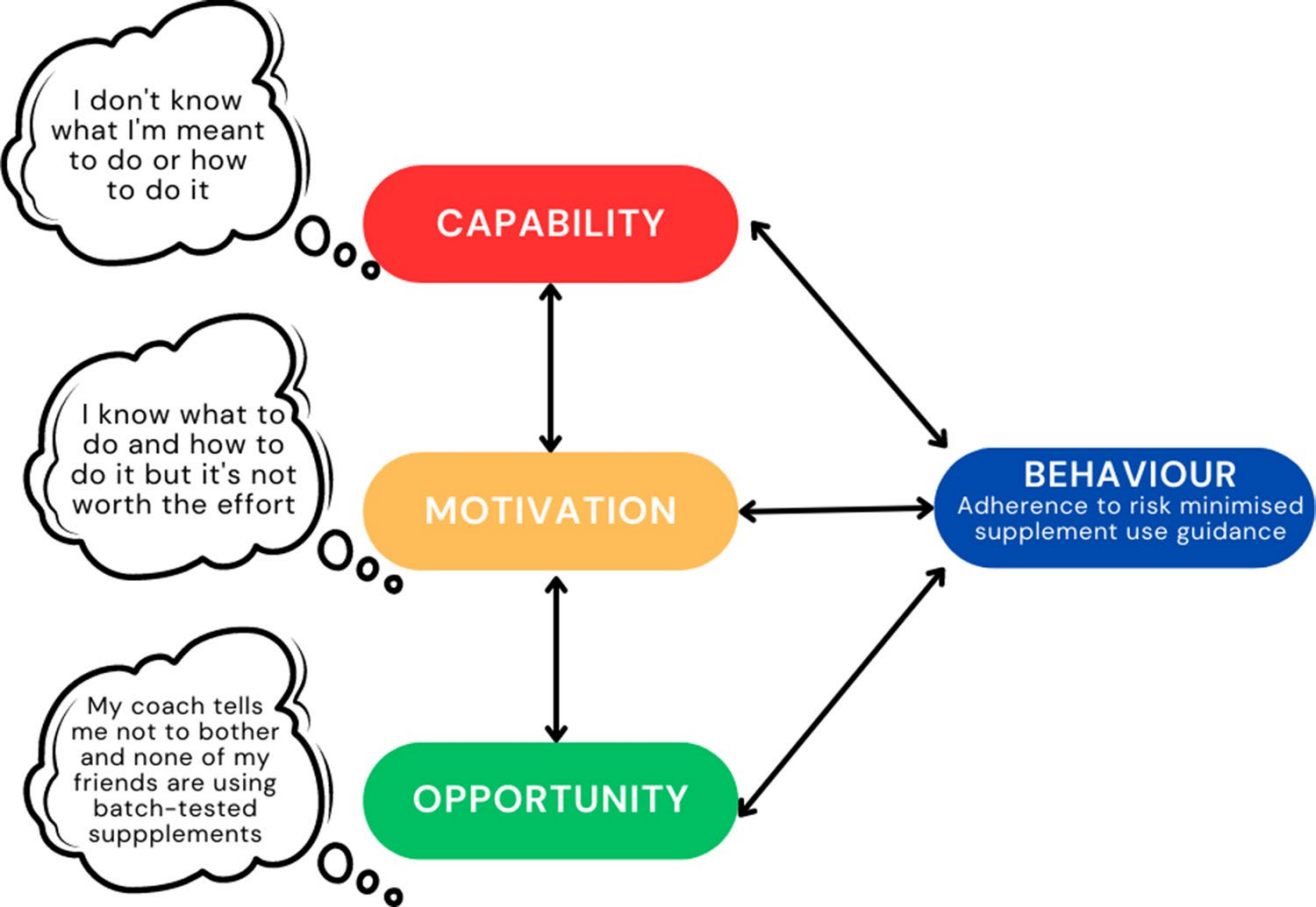
Capability, Opportunity, Motivation-Behaviour Model (COM-B) with adherence to risk minimisation supplement use guidelines (RMSUG) for the target behaviour. Adapted with permission from Michie et al. [63]

At the centre of the BCW is the Capability, Opportunity, Motivation, Behaviour (COM-B) model (the green inner circle in Fig. 3, and shown in more detail in Fig. 6). If we take adherence to RMSUG as the target behaviour in this analysis, the COM-B model [35] recognises that for a competitive athlete to enact this particular behaviour they must have (i) the capability (e.g., knowledge and skills) to ensure the supplements they use are necessary, evidence based, effective (for their sport/context) and free from contaminants/adulterants, (ii) the opportunity to receive assessment/advice from a qualified sports nutritionist/dietitian, and purchase evidence-based, third-party tested supplements (e.g., via easy access to affordable products, supported by their social group) and (iii) the motivation to seek advice, and to only ingest evidence-based, third-party tested supplements over a possibly cheaper and more accessible alternative product that has not been deemed effective or independently tested. All three components are essential, so if any of the components is weak or lacking, adherence to RMSUG has a lower likelihood of occurrence [35].

This narrative review prompts us to consider the modifiable capability, opportunity and motivational barriers to and facilitators of adherence to RMSUG amongst competitive athletes. By appraising current literature in the field related to these components, this narrative review informs recommendations for future actions. Indeed, each of the COM-B components also map across to another framework, the Theoretical Domains Framework (TDF) [64] (Table 1). Providing a synthesis of 33 theories of behaviour and behaviour change clustered into 14 (originally 12) domains [64], the TDF provides a theoretical lens through which to view the cognitive, affective, social and environmental influences on behaviour [65]. Together, the COM-B and TDF provide a comprehensive framework that can be generalised to understand and predict the behaviour of groups, organisations and whole populations. This overarching framework has been used to examine athlete adherence to nutritional guidance [37, 39, 40], explore asthma medication use amongst competitive athletes [41], enhance oral health behaviours in elite athletes [42] and frame a British Association of Sport and Exercise Science Expert Statement on Inadvertent Doping in Sport [66]. These examples recognise that effective service delivery and policy development require an understanding of behaviour that goes beyond common sense [62, 63], and are applying behavioural science to understand and influence behaviour (Fig. 2).

**Table 1:**
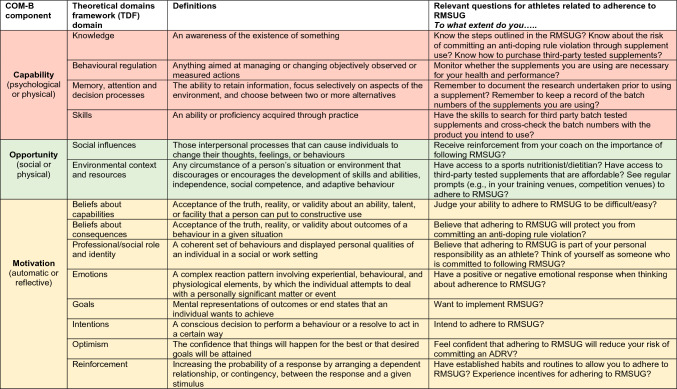
Overview and definition of domains from COM-B and TDF, with example questions related to target behavior (adapted from Michie et al. [62] and Cane et al. [64], with permission)

*ADRV* anti-doping rule violation, *COM-B* Capability, Opportunity, Motivation Model of Behaviour, *RMSUG* risk minimisation supplement use guidelines, *TDF* Theoretical Domains Framework

## Capability

Capability is defined as the individual’s psychological and physical knowledge and skills to engage in an activity [63]. When thinking about the target behaviour of adherence to RMSUG, we might ask whether or not athletes know about the RMSUG and what they are expected to do to reduce the risk of committing an ADRV through supplement use (Table 1). Reviewing the literature, it is apparent that many athletes do not have a clear understanding of critical aspects of supplement use, across many sports and levels of participation (which aligns with the author’s own professional experience). For example, athletes lack knowledge and understanding of the products that are underpinned by a robust evidence base, as well as their active ingredients, mechanisms of action, recommended dose, adverse effects and issues with contamination/adulteration (e.g., [1, 10, 40, 67–72]). Knowledge can therefore serve as both a barrier to and enabler of adherence to RMSUG. For example, athletes who consume supplements without a full understanding or evaluation of the potential benefits and risks associated with their use [1, 69, 73] are not adhering to RMSUG. To illustrate this non-adherence, a study of 574 elite track and field athletes in Japan found there was a gap between the prevalence of supplement use and the evidence supporting the efficacy of the supplements being consumed [11]. In another study involving young athletes from multiple countries (Serbia, Germany, Japan and Croatia), a lack of knowledge about the use of numerous supplements (e.g., creatine, protein) was again noted, yet supplement usage rates were high [69]. Athletes’ lack of knowledge regarding nutritional supplements hinders their capability to assess the *need* for supplements and to judge their effectiveness in their particular context, which serves as a barrier to adherence to RMSUG.

In terms of athletes’ capability to assess the risk of supplement use, and act accordingly, few studies have examined athletes' knowledge of the inherent risks associated with supplementation [1, 15, 44, 69, 74, 75]. However, those studies that have, indicate notable barriers to RMSUG adherence as athletes do not realise the supplements they take can be hazardous [76] and contain prohibited substances [1, 75]. For example, a British rugby player discussed their use of pre-workout supplements, noting that they did not know what was in the product, which was *‘very silly of me’*. They also acknowledged whilst the label specifically indicated it might contain banned substances, they still used the pre-workout supplement (putting themselves at risk of doping) [20]. Another study, conducted in Australia, found that young athletes, when offered an unfamiliar product, did not actively check the ingredients list printed on the package [77]. This is because they were unaware of the need to engage in strategies to avoid unintentional doping in daily life (e.g., reading labels) [77]. The importance of athletes improving their self-monitoring behaviours in order to avoid inadvertent doping has therefore been highlighted [77]. Other studies have noted the challenge of understanding the ingredients lists printed on supplement packaging [78, 79], as well as athletes’ and ASPs unfamiliarity with WADA regulations (e.g., Prohibited List) [2, 52, 69, 74, 80–91]. This gap in knowledge renders athletes vulnerable to inadvertent doping. Therefore, enhancing athlete awareness of the risk of doping when using supplements has been suggested as important to reduce the incidence of doping violations [46]. Further barriers arise from inaccurate understanding of the supplement industry, dis/misinformation associated with the supplement industry [22] as well as athletes’ inaccurate beliefs that supplements are approved by government agencies and tested for safety and efficacy prior to market [76].

Sources of information on supplements for athletes have most commonly been reported as health care professionals, coaches/trainers, the Internet and teammates [17]. Therefore, these significant others have the potential to influence athlete adherence to RMSUG (through their own capability, motivation and opportunity to adhere to RMSUG). Focusing on ASP capability, cross-sectional surveys have demonstrated a lack of knowledge and understanding regarding supplement effects and their associated risks. For example, a study involving over 300 physicians found that about 40% failed to understand that supplements are not approved before they can be sold to consumers [92]. Military physicians also demonstrated insufficient knowledge regarding supplement safety and efficacy [93]. These findings are a cause for concern as elite athletes have acknowledged that they stay away from any form of supplements unless advised and supervised by physicians (who are judged to be ‘experts’) [94]. Coaches also demonstrate a lack of capability to support athletes to adhere to RMSUG and avoid inadvertent doping. Studies have found that coaches believe supplements are essential for athletes’ performance, yet they are unaware of any specific requirements for supplement use [95, 96]. In a rare qualitative study involving nutritionists from Olympic, Paralympic and professional sport, their frustration over the negative influence of the strength and conditioning coach working beyond their professional expertise was apparent. One practitioner noted that myth busting what the strength and conditioning coach had said was “half of their battle”, as it was the opposite to what they wanted the athlete to do [97]. In this study, the technical coach was again seen as a dominant figure who endorses strong cultural beliefs that dispute evidence-based practice and contradict the guidance of the sports nutritionist [97]. In view of these negative social influences, Bentley and colleagues [97] emphasised the importance of engaging key stakeholders in targeted interventions so that they can positively influence athletes’ dietary behaviour during critical moments such as training, recovery and rehabilitation from injury.

Focusing on the behaviour of using third-party tested supplements, knowledge was once again a barrier to initiating and implementing this guidance. For example, in the Netherlands, about half of the Dutch athletes surveyed (*N* = 601) were unfamiliar with the third-party testing system in the Netherlands (NZVT) [74]. Interestingly, females were more familiar with NZVT than males and were more likely to purchase their supplements through the third-party system (81% vs 65%, respectively) [74]. Currently, the study of athletes’ knowledge and understanding of third-party testing systems is lacking in the field [74] and this should be addressed in future research. Third-party testing is essential for elite athletes when purchasing supplements, as it serves to reduce the risk of consumption of contaminated products [2]. However, if athletes (and their ASP) are unaware of the importance of engaging in this process, non-adherence to RMSUG will result.

Given that athletes are vulnerable to inadvertent doping through supplement use, it is important to provide them with the capability to adhere to RMSUG through informed supplement use decision making (e.g., assessing the need, risk and consequence of supplement use). Overall, whilst it is evident that the social, behavioural and environmental antecedents of supplement use represent an underserved research field, it is evident that studies that have explored knowledge of the inherent risks associated with supplement use highlight a lack of knowledge, which acts as a barrier to athlete adherence to RMSUG. If the intervention target is capability, athletes have indicated a preference for ‘effective supplementation’ advice, followed by information about ‘safety or risk concerns’ and then either ‘understanding product labels’ or ‘supplementation benefits’ [15]. Yet, whilst athletes who have received nutritional counselling tended to make better informed choices in relation to supplement use [9], improving nutrition and supplement knowledge alone is not enough to elicit nutritional behaviours [98, 99] and ensure adherence [100] to RMSUG. Interventions need to target all the determinants of behaviour, not just the capability component, which has driven sports nutrition education programmes to date [98].

## Motivation

Motivation is defined as all the brain processes that energise and direct behaviour, not just goals and conscious decision making. A distinction is made between reflective motivational processes (evaluations and plans) and automatic motivational processes (emotions and impulses) [63]. Motivation can serve as both a barrier to and enabler of adherence to RMSUG, depending on the context. Currently, there are no published studies that have specifically examined athletes’ motivation to adhere to RMSUG to prevent inadvertent doping. Whilst research has explored why athletes are attracted to supplements, an extended understanding framed by the multifaceted nature of adherence to RMSUG is required to guide future interventions seeking to reduce the risk of inadvertent doping in at-risk groups. Such research is important if we are to appraise what drives athletes and ASP to adhere to RMSUG, given that a myriad of systemic and individual behavioural determinants must be considered simultaneously. Practically, it is possible to assess an athlete’s motivation to follow RMSUG through a series of questions that can be adapted for athletes and ASP (see Table 1) [62]. Until this specific evidence base is developed, we must tentatively draw upon available research to consider potential motivational determinants of adhering to RMSUG.

An automatic motivational enabler of RMSUG adherence appears to be emotion, which has been identified as a driver of supplement avoidance behaviour in a study of elite athletes [94]. Within this study, an elite athlete explained that they only used natural ingredients out of fear of potential contamination that could lead to inadvertent doping [94]. Fear appeals have been appraised as being effective at positively influencing attitude, intentions and behaviours [101], but their effectiveness in relation to influencing adherence to RMSUG is yet to be established. The significance of reflective motivation as an enabler of RMSUG was illustrated when an athlete, in the same study, stated that *“there are significantly more possibilities to get more out of the body in the so-called grey area or *via* food supplements, *via* shakes, but I tell myself that I don’t need it”* ([94], p. 8). Another athlete also spoke of their religious convictions and identity overriding a desire to use manufactured products [94]. A recent study spanning five European countries further underscored the importance of identity—in this case clean athlete identity [60]—as a potential enabler of adherence to RMSUG. This identity potentially serves as a strong protective mechanism against inadvertent doping through a commitment to following the rules. Research has indicated a general belief that anti-doping policies were a necessary and essential aspect of the effort to maintain ‘clean sport’ [102], but whether this belief transfers to adherence to RMSUG is yet to be determined.

Athletes’ beliefs about supplements and their reasons for use (or non-use) serve as motivational barriers to and enablers of adherence to RMSUG. To date, most of the evidence regarding athlete motives for supplement use has been generated through cross-sectional surveys, but comparisons between surveys are confounded by differences in the use of non-validated and non-standardised survey instruments and the definition of what constitutes a supplement [18]. Still, in a scoping review of 26 articles (including a total of 17,342 athletes), findings were generally consistent, with the most frequently reported reasons for supplement use being improvement of athletic performance, recovery and health [17]. A range of motives have been noted in the literature, and also include more pragmatic reasons such as the convenient provision of energy and nutrients around an exercise session, financial benefits (e.g., sponsorship/provision of free products) and because athletes know or believe that other athletes/competitors are using the supplement(s) [3]. Finally, athletes can be motivated to use supplements as a ‘just in case’ insurance policy [3].

A small number of studies have noted that female athletes are more likely to take supplements for their health while males use them for boosting athletic performance [69]. However, the limited studies available examining sex and gender-based differences render it impossible to draw conclusions at this stage. Studies have also considered the reasons that athletes have decided to not use supplements, with the main reason being a belief that supplements are not required [14, 69, 103, 104]. Correlations have also been reported between greater knowledge of supplements (influencing athletes’ beliefs) and reduced consumption [105]. However, incongruence between supplements used and reasons for using has been highlighted amongst young athletes in the UK and Canada [106, 107]. As such, widespread misinformation regarding supplements and their effects is an issue that needs to be addressed in sport to ensure athletes are using supplements safely and effectively. Failure to do so will serve as a barrier to RMSUG. Further research, drawing on different methodological approaches (e.g., qualitative inquiry), on the motives driving supplement use is also warranted in athletic populations as this deepened understanding will inform interventions to promote adherence to RMSUG.

Alongside significant motivational drivers for supplement use, an obvious and significant barrier to RMSUG is the acknowledgement by consumers that they take supplements through force of habit whereby they do not know if they are actually making any difference. Supplement products are viewed as being fairly benign, with consumers expressing the belief it is better to be safe than sorry [108]. Consequently, research with consumers suggests the perceived benefits of supplement use outweigh the potential costs/risks [2, 3, 13, 17, 80]. This reasoning is compounded by athletes’ beliefs about the consequences of not following RMSUG as there is some evidence of a low perceived threat of detection if prohibited substances are within the supplement they are using. For example, one third of football players had not been tested for drugs within the preceding 2 years, and 60% believe they were unlikely to be tested in the next year [109]. More up-to-date studies are required to comprehensively appraise the threat of detection of prohibited substance use from supplements across multiple sports and competition levels. In sum, athletes will have different motivations for using supplements and adhering (or not) to RMSUG. Further research is now warranted to develop an integrative view of athlete and ASP motivation to adhere to RMSUG.

## Opportunity

Opportunity is defined as all the factors that lie outside the individual and prompt the behaviour (e.g., adherence to RMSUG). Physical opportunity is described as what is physically present within the environment, and social opportunity is described as the cultural milieu or norms that frame thought and decisions [63]. Physical opportunity relates to key features of the physical world which enable or constrain adherence to RMSUG. Taking into account the environmental context, the increasing availability and ease of purchasing supplements which have not been independently tested interact with a lack of knowledge of the inherent risks of a poorly regulated supplement industry (and how to mitigate these risks, as outlined in Sect. 6) and a widespread motivation to use supplements. This interaction renders athletes vulnerable to committing an ADRV. Unlike prescription medications, the safety of supplements is not assessed prior to market [26, 110], and therefore the consumer (in this case, the competitive athlete) is burdened with managing this risk and the consequences, due to the principle of strict liability. In the United States, supplements that are dangerous can only be removed from the market by the Food and Drug Administration (FDA) *after* they have caused harm [26, 111]. Furthermore, supplements are regulated on a national basis and this regulation varies between countries, further exacerbating the risky physical environment for athletes who are trying to adhere to the rules of sport by not consuming prohibited substances [28, 110]. Added to this risky consumer environment is the globally inadequate system for monitoring supplement safety and alerting consumers to emerging risks [21, 26].

The lack of analytical control of supplements before they are introduced to the market, as well as the use of poor manufacturing practices, has led to the availability of many unsafe products [22, 111]. An analysis of the United States FDA warnings from 2007 through 2016 (in the Tainted Supplements database) showed that unapproved pharmaceutical ingredients were identified in 776 supplements listed. These products were commonly marketed for muscle building and weight loss [111], which may be tempting to athletes pursuing performance targets and body composition goals. A recent meta-analysis examining the prevalence of supplement contamination found that almost 28% (875/3132) of the examined supplements posed a potential risk of inadvertent doping (and health harms) due to undeclared substances being found in the products [112]. Therefore, an athlete can unintentionally commit an ADRV without any intention to break the rules. Over the years, studies repeatedly find that many supplements contain undeclared compounds such as prohormones or anabolic androgenic steroids (AAS) [31, 32, 113–121]; the intake of such products significantly increases the risk of inadvertent doping [31, 43]. Most recently, a study conducted in the Netherlands concluded that the problem has not diminished [31]; 38% (25/66 products) of the selected ‘high-risk’ supplements (i.e., those with functional claims of muscle mass gain, increase in fat loss and/or energy boosting) sold online contained undeclared prohibited substances that posed a significant risk of unintentional ADRVs (e.g., stimulants and anabolic steroids). A number of products were also deemed harmful to health.

The complex and dynamic nature of the supplement environment for athletes (as consumers) is illustrated by multiple ADRVs and sanctions arising from athletes who had used supplements containing the prohibited substance higenamine (e.g., Australian marathon runner, Cassie Fien, who was unaware the supplement she was using contained a prohibited substance [122]). Higenamine is predominantly found in supplements marketed for weight loss (11/24; 46%) or energy boosting (11/24) [123]. Added as an example of a β-2 agonist to the WADA Prohibited List in 2017 [123], higenamine is prohibited at all times, both in and out of competition [124]. A natural constituent found in a variety of plant sources and used in traditional botanical remedies, the supplement industry started using it as a substitute for dimethylamylamine (DMAA) and ephedrine [123]. In a study of supplements available for sale in the US, and labelled as containing higenamine (or a synonym, i.e., norcoclaurine or demethylcoclaurine, *Tinospora crispa*) (*N* = 24 products), researchers found them to contain potentially harmful levels of the stimulant and cautioned that the products were inaccurately labelled [123]. Therefore, this study highlights that there is often no way for a consumer to know how much higenamine is actually in the product they are taking, which poses a significant risk to athletes [31]. At the same time, these products are accompanied by bold marketing claims on their packaging and such claims are potentially misleading and lack scientific evidence [125].

The unpredictable nature of the supplement industry presents a considerable risk to the competitive athlete, as they are subject to stringent anti-doping rules. Typically, this risk presents through five different sources [31, 43, 56, 126] whereby the product (i) contains a prohibited substance (whether listed on the ingredient label or not), (ii) lists a prohibited substance on the ingredient label under a different name, (iii) has been cross-contaminated during the manufacturing process, (iv) is a counterfeit product and does not contain the ingredients stated on the label, or (v) has been intentionally adulterated with undeclared substances such as anabolic agents or stimulants in order to increase their efficacy. Better regulation and controls are therefore needed to prevent potential health issues amongst athletes and the general consumer alike [31, 46]. Safety concerns related to the use of certain sports supplements has elicited regulatory and legislative changes in Australia, with a declaration made providing legal clarification on which sports supplements are considered therapeutic goods [127]. Specifically, sports supplements will be regulated by the Australian Government Therapeutic Goods Administration if (a) they are presented in the medicinal dosage form of a pill, tablet or capsule, or (b) their ingredients are of higher risk to consumers [127]. High-risk ingredients in sports supplements are defined as (a) substances present in the product, regardless of how they got there, that are 'scheduled' in the Australian Poisons Standard (e.g., prescription medicine ingredients), (b) ingredients that are intentionally added to the product that are classified as a substance banned for use in sport by WADA and (c) ingredients that are intentionally added to the product that are substances included in a list of 'Relevant Substances' specified in the declaration [127]. Sports supplements that contain ingredients appropriate for food and that are presented as food will be regulated as food (e.g., protein powders, nutrition bars, energy drinks). Australia has taken a decisive pre-market regulatory step to protect the supplement consumer. Further, for the first time in more than a decade, not a single athlete tested positive for taking a supplement according to a Sport Integrity Australia review for 2021–2022 [128]. Despite progress in Australia, a global regulatory shift appears to be some time away [46] and therefore it is critical that immediate action is taken to ensure the physical environment prompts adherence to RMSUG through regular reminders of the importance of adherence to the many behaviours underpinning the RMSUG, as well as easy access to third-party tested supplements, from reputable retailers and at affordable prices.

Prompts could also come from those around the athlete, and social opportunity relates to how the social world enables or constrains adherence to RMSUG, thereby reducing the of committing an ADRV. It is important to acknowledge that supplement use is a socially embedded practice whereby social norms and social influence drive the supplement use behaviours of individuals [46]. Indeed, the increasing social acceptance of consumption of sports supplements [68] presents a greater risk to the consumer. Peers influence the types of supplements used and this could include the promotion of more risky supplements (e.g., weight loss, muscle building) that are not scientifically evidenced and do not offer third-party certification.

In addition to peer influence, the sports nutritionist/dietitian plays a critical role in enabling athletes to adhere to RMSUG [2, 19, 56] and athletes are advised to seek advice from a qualified nutritionist/dietitian prior to using supplements. Yet, many athletes will be unable to follow this advice as they have limited or no access to these qualified professionals [69, 97, 129, 130]. For example, in a study of young athletes across four countries, only 28% of all surveyed athletes had the opportunity to work with dietitians in their sports clubs [69]. As such, athletes face a barrier to adherence as they are unable to seek the necessary advice of a trained professional (and undertake a robust assessment of nutritional needs). Without this professional guidance, athletes may receive supplement information from a wide range of sources, many of whom are not qualified to provide such advice, including team mates, coaches, athletic trainers, family and friends, and the Internet [4–6, 12, 15, 17, 48, 69, 80, 97, 129, 131, 132]. Past research has also evidenced that the media, in the form of magazine and television advertisements, are perceived to be a powerful influence on a person's decision to use supplements [133]. Within the literature available, variability exists with regard to athletes’ self-declared main sources of supplement information.

Regardless of order of influence, the opportunities afforded by the interpersonal interactions with multiple individuals constitute important barriers to and facilitators of adherence to RMSUG [134]. Athletes with access to a sports nutritionist report better informed nutritional supplement choices [9] and/or better dietary habits in general [135]. Athlete–nutritionist relationships based on individual needs, preferences and regular contact seems important. Further, young athletes who used sports supplements ‘properly’ attended more seminars about sports supplementation than others [69]. On the other hand, a lack of information, support and guidance from such trusted professionals, or worse, ignorance and the provision of conflicting information, serve as barriers to adherence and present a significant cause for concern for anti-doping agencies. Despite having access to sports nutritionists and dietitians, some athletes seek advice from less knowledgeable sources [8, 109, 136] and may still choose to use supplements that they do not need, and that have not been batch tested [48, 74]. Indeed, it has been reported that athletes may feel pressured into consuming unfamiliar substances provided by their coaches, team doctors, managers, parents, or other social agents in the sporting context, without questioning the specific ingredient content [78, 79, 137]. As such, athletes may exhibit non-adherence behaviours, but the context within which these behaviours occur can differ significantly. Such contextual heterogeneity needs to be accounted for in future intervention design and development.

## Intervention Design and Development

To prevent inadvertent doping through supplement use, interventions need to be designed with an understanding of what drives athlete and ASP behaviour in relation to RMSUG adherence at a domestic level. This understanding should be framed by a socio-ecological perspective (individual, interpersonal, organisational, community, policy) [138], recognising that athlete behaviour affects, and is affected by, a complex range of social influences and nested environmental interactions. Understanding these interactions at a domestic level through the lens of multiple COM-B analyses will be an important next step for intervention design and development. As a foundation for such work, this review offers a preliminary COM-B analysis of the factors influencing adherence to RMSUG. It has highlighted (i) ‘Capability’-related factors, such as athletes’ and ASPs misconceptions about the safety and regulation of supplement products, lack of knowledge about the risks (e.g., contamination) and efficacy of supplements and their need relative to their sport, and lack of knowledge regarding third-party assurance programmes; (ii) ‘Opportunity’-related factors including lack of access to qualified nutritionists/dietitians, easy access to a growing number of supplement products, lack of regulation of the supplement industry, potential misinformation from influential others (e.g., coaches); and (iii) ‘Motivation’-related factors including supplement use habits, emotions (e.g., fear), clean sport identity, beliefs about the importance of supplement use for performance and perceived threat of detection if a prohibited substance is consumed.

This narrative review has already identified a variety of personal and external factors which influence adherence to RMSUG, and the BCW provides a means by which theoretically underpinned interventions to reduce potential harms from this behaviour could be proposed. Despite the myriad of risks associated with indiscriminate supplement use amongst competitive athletes, to the author’s knowledge there have been no theoretically developed interventions to foster adherence to RMSUG implemented and evaluated in the field. This gap in the knowledge base needs addressing. Early insights from this review point to the importance of interventions that serve to educate, train, persuade and model adherence to RMSUG as normative and as part of an endorsed and preferred sporting culture that protects the integrity and welfare of all. The indiscriminate use of supplements is a concern and requires early intervention for athletes and ASP [3]. More broadly, there is growing concern about the indiscriminate use of self-administered supplements within society at large, as they are often untested and counterproductive to health. Indeed, their use is unlikely to pass a ‘risk–benefit’ appraisal [139]. Therefore, pre-market legislative and regulatory changes are needed to ensure only safe and effective products are available to consumers. However, pragmatism is important as this level of intervention is likely to be a long and slow process, which cannot happen simultaneously around the world [21]. Therefore, we must proactively reduce the risk of inadvertent doping through supplement use via multi-level actions that address multiple interacting factors influencing adherence to RMSUG (e.g., tailored and targeted interventions for athletes and ASP).

## Conclusion

This is the first narrative review to apply a theoretically informed approach to synthesising the evidence base on the barriers to and enablers of adhering to RMSUG. Recognising that supplement use is highly prevalent across sport, despite the risks and consequences that can arise from indiscriminate consumption, this review provides an integrated appraisal of how athletes’ and their support personnel’s capability (knowledge, memory, attention and decision processes), opportunity (physical and social influences) and motivation (beliefs and habits) impact adherence to RMSUG. In doing so, it has highlighted many barriers to and enablers of RMSUG to guide the development of targeted and tailored interventions. However, this review is only the first step towards developing theory- and evidence-based interventions to ensure adherence to risk minimised supplement use behaviour in sport. Future research should identify the intervention types and behaviour change techniques which would be suitable for addressing the factors identified to improve transdisciplinary implementation of comprehensive risk minimised supplement strategies and reduce the risk of inadvertent doping in sport.

## References

[1] Braun H, Koehler K, Geyer H, Kleinert J, Mester J, Schänzer W (2009). Dietary supplement use among elite young German athletes. Int J Sport Nutr Exerc Metab.

[2] Maughan RJ, Burke LM, Dvorak J, Larson-Meyer DE, Peeling P, Phillips SM (2018). IOC consensus statement: dietary supplements and the high-performance athlete. Br J Sports Med.

[3] Garthe I, Maughan RJ (2018). Athletes and supplements: prevalence and perspectives. Int J Sport Nutr Exerc Metab.

[4] Froiland K, Koszewski W, Hingst J, Kopecky L (2004). Nutritional supplement use among college athletes and their sources of information. Int J Sport Nutr Exerc Metab.

[5] Diehl K, Thiel A, Zipfel S, Mayer J, Schnell A, Schneider S (2012). Elite adolescent athletes’ use of dietary supplements: characteristics, opinions, and sources of supply and information. Int J Sport Nutr Exerc Metab.

[6] Knapik JJ, Steelman RA, Hoedebecke SS, Austin KG, Farina EK, Lieberman HR (2016). Prevalence of dietary supplement use by athletes: systematic review and meta-analysis. Sports Med.

[7] Kim J, Kang S, Jung H, Chun Y, Trilk J, Jung SH (2011). Dietary supplementation patterns of Korean olympic athletes participating in the Beijing 2008 Summer Olympic Games. Int J Sport Nutr Exerc Metab.

[8] Baltazar-Martins G, Brito de Souza D, Aguilar-Navarro M, Muñoz-Guerra J, Plata M del M, Del Coso J. Prevalence and patterns of dietary supplement use in elite Spanish athletes. J Int Soc Sports Nutr. 2019;16:1–9. 10.1186/s12970-019-0296-5 PMC6639916 31319850

[9] Wardenaar FC, Ceelen IJM, Van Dijk J-W, Hangelbroek RWJ, Van Roy L, Van der Pouw B (2017). Nutritional supplement use by Dutch elite and sub-elite athletes: does receiving dietary counseling make a difference?. Int J Sport Nutr Exerc Metab.

[10] Sato A, Kamei A, Kamihigashi E, Dohi M, Komatsu Y, Akama T (2012). Use of supplements by young elite Japanese athletes participating in the 2010 youth Olympic games in Singapore. Clin J Sport Med.

[11] Tabata S, Yamasawa F, Torii S, Manabe T, Kamada H, Namba A (2020). Use of nutritional supplements by elite Japanese track and field athletes. J Int Soc Sports Nutr.

[12] Sato A, Kamei A, Kamihigashi E, Dohi M, Akama T, Kawahara T (2015). Use of supplements by Japanese elite athletes for the 2012 Olympic Games in London. Clin J Sport Med.

[13] Wiens K, Erdman KA, Stadnyk M, Parnell JA (2014). Dietary supplement usage, motivation, and education in young Canadian athletes. Int J Sport Nutr Exerc Metab.

[14] Nieper A (2005). Nutritional supplement practices in UK junior national track and field athletes. Br J Sports Med.

[15] Erdman KA, Fung TS, Reimer RA (2006). Influence of performance level on dietary supplementation in elite Canadian athletes. Med Sci Sports Exerc.

[16] Slater G, Tan B, Teh KC (2003). Dietary supplementation practices of Singaporean athletes. Int J Sport Nutr Exerc Metab.

[17] Daher J, Mallick M, El Khoury D (2022). Prevalence of dietary supplement use among athletes worldwide: a scoping review. Nutrients.

[18] Garthe I, Ramsbottom R (2020). Elite athletes, a rationale for the use of dietary supplements: a practical approach. PharmaNutrition.

[19] Close GL, Kasper AM, Walsh NP, Maughan RJ (2022). “Food first but not always food only”: recommendations for using dietary supplements in sport. Int J Sport Nutr Exerc Metab.

[20] Didymus FF, Backhouse SH (2020). Coping by doping? A qualitative inquiry into permitted and prohibited substance use in competitive rugby. Psychol Sport Exerc.

[21] Dwyer JT, Coates PM, Smith MJ (2018). Dietary supplements: regulatory challenges and research resources. Nutrients.

[22] Tiller NB, Sullivan JP, Ekkekakis P (2023). Baseless claims and pseudoscience in health and wellness: a call to action for the sports, exercise, and nutrition-science community. Sports Med.

[23] Starr RR (2015). Too little, too late: ineffective regulation of dietary supplements in the United States. Am J Public Health.

[24] Peeling P, Binnie MJ, Goods PSR, Sim M, Burke LM (2018). Evidence-based supplements for the enhancement of athletic performance. Int J Sport Nutr Exerc Metab.

[25] Australian Institute for Sport. Australian Institute of Sport (AIS) Position Statement: supplements and sports foods in high performance sport [Internet]; 2022. https://www.ais.gov.au/__data/assets/pdf_file/0014/1000841/Position-Statement-Supplements-and-Sports-Foods-abridged_v2.pdf [cited 1 Oct 2022].

[26] Cohen PA (2014). Hazards of hindsight—monitoring the safety of nutritional supplements. N Engl J Med.

[27] Chatham-Stephens K, Taylor E, Chang A, Peterson A, Daniel J, Martin C (2017). Hepatotoxicity associated with weight loss or sports dietary supplements, including OxyELITE Pro™—United States, 2013. Drug Test Anal.

[28] Geller AI, Shehab N, Weidle NJ, Lovegrove MC, Wolpert BJ, Timbo BB (2015). Emergency department visits for adverse events related to dietary supplements. N Engl J Med.

[29] Parr MK, Geyer H, Hoffmann B, Köhler K, Mareck U, Schänzer W (2007). High amounts of 17-methylated anabolic-androgenic steroids in effervescent tablets on the dietary supplement market. Biomed Chromatogr.

[30] Geyer H, Bredehöft M, Mareck U, Parr M, Schänzer W (2003). High doses of the anabolic steroid metandienone found in dietary supplements. Eur J Sport Sci.

[31] Duiven E, van Loon LJC, Spruijt L, Koert W, de Hon OM (2021). Undeclared doping substances are highly prevalent in commercial sports nutrition supplements. J Sports Sci Med.

[32] Kamber M, Baume N, Saugy M, Rivier L (2001). Nutritional supplements as a source for positive doping cases?. Int J Sport Nutr Exerc Metab.

[33] Whitaker L, Backhouse S (2017). Doping in sport: an analysis of sanctioned UK rugby union players between 2009 and 2015. J Sports Sci.

[34] Backhouse S, Whitaker L (2016). Nutritional supplements in sport: prevalence, reasons for use, and relation to doping. Psychol Doping Sport.

[35] Knapik JJ, Trone DW, Steelman RA, Farina EK, Lieberman HR (2022). Adverse effects associated with use of specific dietary supplements: the US Military Dietary Supplement Use Study. Food Chem Toxicol Int J Publ Br Ind Biol Res Assoc.

[36] Stickel F, Kessebohm K, Weimann R, Seitz HK (2011). Review of liver injury associated with dietary supplements. Liver Int.

[37] Avelar-Escobar G, Méndez-Navarro J, Ortiz-Olvera NX, Castellanos G, Ramos R, Gallardo-Cabrera VE (2012). Hepatotoxicity associated with dietary energy supplements: use and abuse by young athletes. Ann Hepatol.

[38] Backhouse SH, Whitaker L, Petróczi A (2013). Gateway to doping? Supplement use in the context of preferred competitive situations, doping attitude, beliefs, and norms. Scand J Med Sci Sports.

[39] Hurst P, Kavussanu M, Boardley I, Ring C (2019). Sport supplement use predicts doping attitudes and likelihood via sport supplement beliefs. J Sports Sci.

[40] Mathews NM (2018). Prohibited contaminants in dietary supplements. Sports Health Multidiscip Approach.

[41] Ntoumanis N, Ng JYY, Barkoukis V, Backhouse S (2014). Personal and psychosocial predictors of doping use in physical activity settings: a meta-analysis. Sports Med.

[42] Hurst P, Schiphof-Godart L, Kavussanu M, Barkoukis V, Petróczi A, Ring C (2023). Are dietary supplement users more likely to dope than non-users? A systematic review and meta-analysis. Int J Drug Policy.

[43] Lauritzen F. Dietary supplements as a major cause of anti-doping rule violations. Front Sports Act Living. 2022;4:101. 10.3389/fspor.2022.868228 PMC8990797 35399596

[44] Baylis A, Cameron-Smith D, Burke LM (2001). Inadvertent doping through supplement use by athletes: assessment and management of the risk in Australia. Int J Sport Nutr Exerc Metab.

[45] Outram S, Stewart B (2015). Doping through supplement use: a review of the available empirical data. Int J Sport Nutr Exerc Metab.

[46] Backhouse, S.H., Duiven, E., Staff, H., Bentley, M. Reducing the risk of inadvertent doping from food supplement use: current practice and future actions. [Internet]. Brussels: EuropeActive; 2019. https://ec.europa.eu/programmes/erasmus-plus/project-result-content/5d7860db-eda6-4a62-830b-f5958c292e67/FAIR_Final_Report.pdf.

[47] Eichner AK, Coyles J, Fedoruk M, Maxey TD, Lenaghan RA, Novitzky J (2019). Essential features of third-party certification programs for dietary supplements: a consensus statement. Curr Sports Med Rep.

[48] Vento KA, Wardenaar FC (2020). Third-party testing nutritional supplement knowledge, attitudes, and use among an NCAA I collegiate student-athlete population. Front Sports Act Living.

[49] World Anti-Doping Agency. World Anti-Doping Code [Internet]. World Anti-Doping Agency. https://www.wada-ama.org/en/resources/world-anti-doping-program/world-anti-doping-code [cited 19 Sep 2022].

[50] McArdle D. ‘Strict liability’ and legal rights: nutritional supplements, ‘intent’ and ‘risk’ in the parallel world of WADA. Routledge Handb Drugs Sport. London: Routledge/Taylor & Francis Group; 2015.

[51] Overbye M, Elbe A-M, Knudsen ML, Pfister G (2015). Athletes’ perceptions of anti-doping sanctions: the ban from sport versus social, financial and self-imposed sanctions. Sport Soc.

[52] Qvarfordt A, Ahmadi N, Bäckström Å, Hoff D (2021). Limitations and duties: elite athletes’ perceptions of compliance with anti-doping rules. Sport Soc.

[53] Athletics Integrity Unit. AIU URGES VIGILANCE AMID UJAH’S BAN [Internet]; 2022. https://www.athleticsintegrity.org/downloads/pdfs/other/Press-Release-Ujah.pdf [cited 27 Oct 2022].

[54] Ingle S. Banned British sprinter CJ Ujah cleared of deliberately taking drugs at Olympics. The Guardian [Internet]. 2022 Oct 10. https://www.theguardian.com/sport/2022/oct/10/banned-british-sprinter-cj-ujah-cleared-of-deliberately-taking-drugs-at-olympics [cited 27 Oct 2022].

[55] Kassel MA, From A (1994). History of near misses: the future of dietary supplement regulation. Food Drug Law J.

[56] British Dietetics Association Sports Nutrition Group. Nutritional Supplement Position Statement [Internet]; 2022. https://www.bda.uk.com/uploads/assets/b7c89c32-40d8-4f66-9449aae9fc8c81cc/SNG-Supplement-Position-Statement-2022.pdf [cited 19 Dec 2022].

[57] Chakrabarti S (2014). What’s in a name? Compliance, adherence and concordance in chronic psychiatric disorders. World J Psychiatry.

[58] US Anti-Doping Agency. Supplement Guide: Reducing Supplement Risk [Internet]. https://www.usada.org/wp-content/uploads/supplement-guide.pdf [cited 17 Apr 2023].

[59] Fortington LV, Handcock RN, Derman W, Emery CA, Pasanen K, Schwellnus M (2023). Citation impact and reach of the IOC sport and exercise medicine consensus statements. BMJ Open Sport Exerc Med.

[60] Petróczi A, Heyes A, Thrower SN, Martinelli LA, Backhouse SH, Boardley ID (2021). Understanding and building clean(er) sport together: community-based participatory research with elite athletes and anti-doping organisations from five European countries. Psychol Sport Exerc.

[61] Backhouse SH, Griffiths C, McKenna J (2018). Tackling doping in sport: a call to take action on the dopogenic environment. Br J Sports Med.

[62] Michie S, Atkins L, West R. The behaviour change wheel book—a guide to designing interventions [Internet]. Silverback Publishing; 2014. http://www.behaviourchangewheel.com/ [cited 13 Feb 2023].

[63] Michie S, van Stralen MM, West R (2011). The behaviour change wheel: a new method for characterising and designing behaviour change interventions. Implement Sci.

[64] Cane J, O’Connor D, Michie S (2012). Validation of the theoretical domains framework for use in behaviour change and implementation research. Implement Sci IS.

[65] Atkins L, Francis J, Islam R, O’Connor D, Patey A, Ivers N (2017). A guide to using the Theoretical Domains Framework of behaviour change to investigate implementation problems. Implement Sci.

[66] Backhouse S, Boardley I, Chester N, Currell K, Hudson A, Mills K, et al. The BASES expert statement on inadvertent doping in sport [Internet]. British Association of Sport and Exercise Sciences; 2017. https://www.bases.org.uk/imgs/expert_statement_winter_2017__revise_2_201.pdf [cited 22 Oct 2022].

[67] Jessri M, Jessri M, RashidKhani B, Zinn C (2010). Evaluation of Iranian college athletes’ sport nutrition knowledge. Int J Sport Nutr Exerc Metab.

[68] Dascombe BJ, Karunaratna M, Cartoon J, Fergie B, Goodman C (2010). Nutritional supplementation habits and perceptions of elite athletes within a state-based sporting institute. J Sci Med Sport.

[69] Jovanov P, Đorđić V, Obradović B, Barak O, Pezo L, Marić A (2019). Prevalence, knowledge and attitudes towards using sports supplements among young athletes. J Int Soc Sports Nutr.

[70] Torres-McGehee TM, Pritchett KL, Zippel D, Minton DM, Cellamare A, Sibilia M (2012). Sports nutrition knowledge among collegiate athletes, coaches, athletic trainers, and strength and conditioning specialists. J Athl Train.

[71] Jonnalagadda SS, Rosenbloom CA, Skinner R (2001). Dietary practices, attitudes, and physiological status of collegiate freshman football players. J Strength Cond Res.

[72] Jagim AR, Camic CL, Harty PS (2019). Common habits, adverse events, and opinions regarding pre-workout supplement use among regular consumers. Nutrients.

[73] Maughan RJ, Depiesse F, Geyer H, International Association of Athletics Federations (2007). The use of dietary supplements by athletes. J Sports Sci.

[74] Wardenaar FC, Hoogervorst D, Vento KA, de Hon PhDO (2021). Dutch olympic and non-olympic athletes differ in knowledge of and attitudes toward third-party supplement testing. J Diet Suppl.

[75] Lamont-Mills A, Christensen S (2008). “I have never taken performance enhancing drugs and I never will”: drug discourse in the Shane Warne case. Scand J Med Sci Sports.

[76] Dodge T (2016). Consumers’ perceptions of the dietary supplement health and education act: implications and recommendations. Drug Test Anal.

[77] Chan DKC, Donovan RJ, Lentillon-Kaestner V, Hardcastle SJ, Dimmock JA, Keatley DA (2015). Young athletes’ awareness and monitoring of anti-doping in daily life: does motivation matter?. Scand J Med Sci Sports.

[78] Chan DKC, Hardcastle SJ, Lentillon-Kaestner V, Donovan RJ, Dimmock JA, Hagger MS (2014). Athletes’ beliefs about and attitudes towards taking banned performance-enhancing substances: a qualitative study. Sport Exerc Perform Psychol.

[79] Johnson J, Butryn T, Masucci M (2013). A focus group analysis of the US and Canadian female triathletes’ knowledge of doping. Sport Soc.

[80] Sekulic D, Tahiraj E, Maric D, Olujic D, Bianco A, Zaletel P (2019). What drives athletes toward dietary supplement use: objective knowledge or self-perceived competence? Cross-sectional analysis of professional team-sport players from Southeastern Europe during the competitive season. J Int Soc Sports Nutr.

[81] Mazanov J, Backhouse S, Connor J, Hemphill D, Quirk F (2014). Athlete support personnel and anti-doping: knowledge, attitudes, and ethical stance. Scand J Med Sci Sports.

[82] Backhouse SH, McKenna J (2012). Reviewing coaches’ knowledge, attitudes and beliefs regarding doping in sport. Int J Sports Sci Coach.

[83] Engelberg T, Moston S (2016). Inside the locker room: a qualitative study of coaches’ anti-doping knowledge, beliefs and attitudes. Sport Soc.

[84] Morente-Sánchez J, Zabala M (2015). Knowledge, attitudes and beliefs of technical staff towards doping in Spanish football. J Sports Sci.

[85] Muwonge H, Zavuga R, Kabenge PA (2015). Doping knowledge, attitudes, and practices of Ugandan athletes’: a cross-sectional study. Subst Abuse Treat Prev Policy.

[86] Mottram D, Khalifa S, Alemrayat B, Rahhal A, Ahmed A, Stuart M (2016). Perspective of pharmacists in Qatar regarding doping and anti-doping in sports. J Sports Med Phys Fit.

[87] Murofushi Y, Kawata Y, Kamimura A, Hirosawa M, Shibata N (2018). Impact of anti-doping education and doping control experience on anti-doping knowledge in Japanese university athletes: a cross-sectional study. Subst Abuse Treat Prev Policy.

[88] Greenbaum DH, McLachlan AJ, Roubin RH, Moles R, Chaar BB (2023). Examining pharmacists’ anti-doping knowledge and skills in assisting athletes to avoid unintentional use of prohibited substances. Int J Pharm Pract.

[89] Mottram D, Chester N, Atkinson G, Goode D. Athletes’ knowledge and views on OTC medication. Int J Sports Med. 2008;851–5. 10.1055/s-2008-1038403 18401811

[90] Voravuth N, Chua EW, Mahmood TMT, Lim MC, Puteh SEW, Safii NS (2022). Engaging community pharmacists to eliminate inadvertent doping in sports: a study of their knowledge on doping. PLoS One.

[91] Patterson LB, Backhouse SH, Lara-Bercial S (2019). Examining coaches’ experiences and opinions of anti-doping education. Int Sport Coach J.

[92] Dodge T, Litt D, Kaufman A (2011). Influence of the dietary supplement health and education act on consumer beliefs about the safety and effectiveness of dietary supplements. J Health Commun.

[93] Cellini M, Attipoe S, Seales P, Gray R, Ward A, Stephens M (2013). Dietary supplements: physician knowledge and adverse event reporting. Med Sci Sports Exerc.

[94] Clancy S, Owusu-Sekyere F, Shelley J, Veltmaat A, De Maria A, Petróczi A (2022). The role of personal commitment to integrity in clean sport and anti-doping. Perform Enhanc Health.

[95] Cherian KS, Gavaravarapu SM, Sainoji A, Yagnambhatt VR (2020). Coaches’ perceptions about food, appetite, and nutrition of adolescent Indian athletes—a qualitative study. Heliyon.

[96] Seif Barghi T, Halabchi F, Dvorak J, Hosseinnejad H (2015). How the Iranian football coaches and players know about doping?. Asian J Sports Med.

[97] Bentley MR, Mitchell N, Sutton L, Backhouse SH (2019). Sports nutritionists’ perspectives on enablers and barriers to nutritional adherence in high performance sport: a qualitative analysis informed by the COM-B model and theoretical domains framework. J Sports Sci.

[98] Bentley MRN, Mitchell N, Backhouse SH (2020). Sports nutrition interventions: a systematic review of behavioural strategies used to promote dietary behaviour change in athletes. Appetite.

[99] Sánchez-Díaz S, Yanci J, Castillo D, Scanlan AT, Raya-González J (2020). Effects of nutrition education interventions in team sport players. A systematic review. Nutrients.

[100] West R, Gould A. Improving health and wellbeing: A guide to using behavioural science in policy and practice [Internet]; 2022. https://phwwhocc.co.uk/wp-content/uploads/2022/11/A-Guide-to-Using-Behavioural-Science_ENGLISH.pdf.

[101] Tannenbaum MB, Hepler J, Zimmerman RS, Saul L, Jacobs S, Wilson K (2015). Appealing to fear: a meta-analysis of fear appeal effectiveness and theories. Psychol Bull.

[102] Woolway T, Lazuras L, Barkoukis V, Petróczi A (2020). “Doing what is right and doing it right”: a mapping review of athletes’ perception of anti-doping legitimacy. Int J Drug Policy.

[103] Nabuco HCG, Rodrigues VB, Barros WM de, Ravagnani FC de P, Espinosa MM, Ravagnani C de FC. Use of dietary supplements among Brazilian athletes. Rev Nutr. 2017;30:163–73.

[104] Tawfik S, Koofy NE, Moawad EMI (2016). Patterns of nutrition and dietary supplements use in young egyptian athletes: a community-based cross-sectional survey. PLoS One.

[105] Bianco A, Mammina C, Paoli A, Bellafiore M, Battaglia G, Caramazza G (2011). Protein supplementation in strength and conditioning adepts: knowledge, dietary behavior and practice in Palermo, Italy. J Int Soc Sports Nutr.

[106] Petróczi A, Naughton DP, Pearce G, Bailey R, Bloodworth A, McNamee M (2008). Nutritional supplement use by elite young UK athletes: fallacies of advice regarding efficacy. J Int Soc Sports Nutr.

[107] Parnell JA, Wiens K, Erdman KA (2015). Evaluation of congruence among dietary supplement use and motivation for supplementation in young, Canadian athletes. J Int Soc Sports Nutr.

[108] Food Standards Agency. Food supplements consumer research [Internet]. Food Stand. Agency; 2018. https://www.food.gov.uk/research/behaviour-and-perception/food-supplements-consumer-research [cited 27 Feb 2022].

[109] Waddington I, Malcolm D, Roderick M, Naik R (2005). Drug use in English professional football. Br J Sports Med.

[110] Cohen PA (2018). The FDA and adulterated supplements—dereliction of duty. JAMA Netw Open.

[111] Tucker J, Fischer T, Upjohn L, Mazzera D, Kumar M (2018). Unapproved pharmaceutical ingredients included in dietary supplements associated with US food and drug administration warnings. JAMA Netw Open.

[112] Kozhuharov VR, Ivanov K, Ivanova S. Dietary supplements as source of unintentional doping. BioMed Res Int. 2022;1–18. 10.1155/2022/8387271 PMC9054437 35496041

[113] Pascali J, Piva E, Forcato M, Boscolo-Berto R, Rondinelli R, Fais P (2022). Detection of multiple prohibited ingredients in nutritional supplements in a case of doping. Toxicol Anal Clin.

[114] Geyer H, Parr MK, Koehler K, Mareck U, Schänzer W, Thevis M (2008). Nutritional supplements cross-contaminated and faked with doping substances. J Mass Spectrom.

[115] Green GA, Catlin DH, Starcevic B (2001). Analysis of over-the-counter dietary supplements. Clin J Sport Med.

[116] Van Poucke C, Detavernier C, Van Cauwenberghe R, Van Peteghem C (2007). Determination of anabolic steroids in dietary supplements by liquid chromatography–tandem mass spectrometry. Anal Chim Acta.

[117] Stepan R, Cuhra P, Barsova S (2008). Comprehensive two-dimensional gas chromatography with time-of-flight mass spectrometric detection for the determination of anabolic steroids and related compounds in nutritional supplements. Food Addit Contam Part A.

[118] Abbate V, Kicman AT, Evans-Brown M, McVeigh J, Cowan DA, Wilson C (2015). Anabolic steroids detected in bodybuilding dietary supplements—a significant risk to public health. Drug Test Anal.

[119] Rijk JCW, Bovee TFH, Wang S, Van Poucke C, Van Peteghem C, Nielen MWF (2009). Detection of anabolic steroids in dietary supplements: The added value of an androgen yeast bioassay in parallel with a liquid chromatography–tandem mass spectrometry screening method. Anal Chim Acta.

[120] De Cock KJS, Delbeke FT, Van Eenoo P, Desmet N, Roels K, De Backer P (2001). Detection and determination of anabolic steroids in nutritional supplements. J Pharm Biomed Anal.

[121] Leaney AE, Beck P, Biddle S, Brown P, Grace PB, Hudson SC (2021). Analysis of supplements available to UK consumers purporting to contain selective androgen receptor modulators. Drug Test Anal.

[122] Sport Integrity Australia. Supplements: A cautionary tale | Sport Integrity Australia [Internet]. Suppl. CAUTIONARY TALE; 2020. https://www.sportintegrity.gov.au/news/integrity-blog/2020-04/supplements-cautionary-tale [cited 13 Mar 2021].

[123] Cohen PA, Travis JC, Keizers PHJ, Boyer FE, Venhuis BJ (2019). The stimulant higenamine in weight loss and sports supplements. Clin Toxicol.

[124] World Anti-Doping Agency. WADA Prohibited List [Internet]; 2023. https://www.wada-ama.org/sites/default/files/2022-09/2023list_en_final_9_september_2022.pdf [cited 10 Oct 2023].

[125] Hua SV, Granger B, Bauer K, Roberto CA (2021). A content analysis of marketing on the packages of dietary supplements for weight loss and muscle building. Prev Med Rep.

[126] Martínez-Sanz JM, Sospedra I, Mañas Ortiz C, Baladía E, Gil-Izquierdo A, Ortiz-Moncada R (2017). Intended or unintended doping? A review of the presence of doping substances in dietary supplements used in sports. Nutrients.

[127] Australian Government Therapeutic Goods Administration. Sports supplements declared to be medicines: Consumer fact sheet [Internet]. Ther. Goods Adm. TGA. Therapeutic Goods Administration (TGA); 2020. https://www.tga.gov.au/news/news/sports-supplements-declared-be-medicines [cited 18 Oct 2022].

[128] Sport Integrity Australia. Annual Report 2021–22. https://www.sportintegrity.gov.au/sites/default/files/SIA003-0722_ANNUAL%20REPORT_WEB_ACCESSIBLE3.pdf [cited 22 Jan 2023].

[129] Burns RD, Schiller MR, Merrick MA, Wolf KN (2004). Intercollegiate student athlete use of nutritional supplements and the role of athletic trainers and dietitians in nutrition counseling. J Am Diet Assoc.

[130] Vento KA, Delgado F, Skinner J, Wardenaar FC. Funding and college-provided nutritional resources on diet quality among female athletes. J Am Coll Health. 2021;1–8. 10.1080/07448481.2021.1947301 34379567

[131] Graham-Paulson TS, Perret C, Smith B, Crosland J, Goosey-Tolfrey VL (2015). Nutritional supplement habits of athletes with an impairment and their sources of information. Int J Sport Nutr Exerc Metab.

[132] Bentley MRN, Patterson LB, Mitchell N, Backhouse SH (2021). Athlete perspectives on the enablers and barriers to nutritional adherence in high-performance sport. Psychol Sport Exerc.

[133] Conner M, Kirk SF, Cade JE, Barrett JH (1982). Why do women use dietary supplements? The use of the theory of planned behaviour to explore beliefs about their use. Soc Sci Med.

[134] Wardenaar FC, Hoogervorst D (2022). How sports health professionals perceive and prescribe nutritional supplements to Olympic and non-Olympic athletes. Int J Environ Res Public Health.

[135] Hull MV, Jagim AR, Oliver JM, Greenwood M, Busteed DR, Jones MT (2016). Gender differences and access to a sports dietitian influence dietary habits of collegiate athletes. J Int Soc Sports Nutr.

[136] Heikkinen A, Alaranta A, Helenius I, Vasankari T (2011). Dietary supplementation habits and perceptions of supplement use among elite Finnish athletes. Int J Sport Nutr Exerc Metab.

[137] Ntoumanis N, Barkoukis V, Gucciardi DF, Chan DKC (2017). Linking coach interpersonal style with athlete doping intentions and doping use: a prospective study. J Sport Exerc Psychol.

[138] Bronfenbrenner U (1977). Toward an experimental ecology of human development. Am Psychol.

[139] Campbell A, Carins J, Rundle-Thiele S, Deshpande S, Baker B (2021). Motivators of indiscriminate and unsafe supplement use among young Australians. Int J Environ Res Public Health.

